# Psychological Burden Among Patients With Inherited Bleeding Disorders in Madinah Province, Saudi Arabia

**DOI:** 10.7759/cureus.45165

**Published:** 2023-09-13

**Authors:** Mohammed A Zolaly, Ali H Alshawi, Idris A Binsari, Abdullah A Alharbi, Yazeed N Almutairi, Fayzah M Zolaly, Mohammed A Al Belowi

**Affiliations:** 1 Hematology and Oncology, Taibah University, Al-Madinah al-Munawwarah, SAU; 2 Pediatrics, Taibah University, Al-Madinah al-Munawwarah, SAU; 3 Internal Medicine/Hematology, Taibah University, Al-Madinah al-Munawwarah, SAU

**Keywords:** inherited bleeding disorders, kingdom of saudi arabia (ksa), stress, anxiety levels, depression

## Abstract

Introduction

Hereditary bleeding disorders, such as hemophilia and von Willebrand disease (VWD), result from specific deficiencies or malformations in the coagulation cascade proteins. These disorders can significantly impact both physical and psychological health. Complications such as depression, anxiety, and stress (DAS) can further exacerbate these impacts. Despite their significance, detailed prevalence data remain limited, especially for regions such as Madinah province in Saudi Arabia. This study aimed to determine the prevalence of DAS and their associated risk factors among patients with hereditary bleeding disorders in Madinah province, Saudi Arabia.

Methods

We conducted a cross-sectional study using telephonic interviews involving patients diagnosed with severe hemophilia A or B or VWD attending a hematology clinic in Madinah. Patients over 10 were included, and the study excluded those with central nervous system insults and platelet count concerns. The validated and reliable Depression Anxiety Stress Scale-21-item questionnaire and Statistical Product and Service Solutions (SPSS), version 26.0 (IBM SPSS Statistics for Windows, Armonk, NY), facilitated data collection and analysis, respectively.

Results

Of the 44 patients studied, 25% exhibited symptoms of depression, 45.5% showed signs of anxiety, and 29.5% had stress symptoms. Regarding symptom severity, 9.1% of patients experienced extremely severe depression, 15.9% had moderate anxiety, and 13.6% reported moderate stress. The prevalence of these psychological issues varied with patients' age and economic status. Notably, a significantly higher rate of depression was observed in patients over 15 years (42.9% vs. 8.7%; p=0.009). Additionally, while not statistically significant, patients with a high economic status reported increased rates of DAS.

Conclusions

Patients with inherited bleeding disorders, particularly those older than 15, manifest significant psychological distress. There is a pressing need for enhanced awareness, specialized screenings, and tailored counseling services to improve treatment adherence and overall quality of life. Given the findings, a comprehensive national study in Saudi Arabia is highly recommended, alongside the integration of specialized psychological services.

## Introduction

Hereditary bleeding disorders arise due to a decrease or absence of specific clotting proteins in the coagulation cascade. The three most common inherited bleeding disorders are factor VIII deficiency (hemophilia A), factor IX deficiency (hemophilia B), and von Willebrand disease (VWD) [1]. Hemophilia A affects roughly one in 10,000 males, whereas hemophilia B's prevalence is approximately one in 35,000-50,000 males. Regrettably, current data do not provide a precise prevalence for our country, Saudi Arabia [2].

The signs and symptoms of hemophilia vary based on disease severity, which correlates with factor levels. Patients with mild hemophilia often present symptoms later in childhood. In contrast, severe cases might manifest as early as the neonatal period, with most diagnoses occurring within the first year of life. Factor levels can be influenced by both age and the patient's blood group. Thus, it is crucial to account for physiological variations in factor levels when diagnosing factor deficiency. If not appropriately managed, hemophiliac patients can develop severe complications, including joint deformities that limit movement, contractures, chronic pain, muscle atrophy, and significant neurological issues [2].

von Willebrand factor, a glycoprotein, plays a crucial role in both primary hemostasis and the intrinsic pathway of secondary hemostasis. It is the most prevalent congenital bleeding disorder globally, impacting both sexes since it follows an autosomal inheritance pattern. Depending on the disease type, patients might exhibit a total absence, partial absence, or functional anomaly of the von Willebrand factor [3,4].

All hereditary bleeding disorders can inflict considerable psychological strain on both patients and their families. Symptoms such as depression, anxiety, and stress often stem from the disease's chronic nature and its associated physical complications. The ensuing financial hardships, potential unemployment, and frequent school absences or failures can further deteriorate their psychological health [5]. This study aimed to determine the prevalence and associated risk factors of depression, anxiety, and stress among patients with hereditary bleeding disorders in Madinah Province, Saudi Arabia.

## Materials and methods

We conducted a cross-sectional study using questionnaires via individual telephonic interviews with patients and their families. Interviews were initiated after securing approval from the Taibah University Research Ethics Board Committee (Approval No. STU-21-016).

We included patients older than 10, diagnosed with severe hemophilia A or B or VWD, who were registered with the Madinah Hereditary Blood Disorders Charity Society in the year 2022, resided in Madinah Province, and spoke Arabic. Patients were only included after providing informed consent. We excluded patients with a central nervous system insult, either from a prior bleed or another disease, and patients with platelet count issues or functional disorders. The questionnaire captured demographic information, such as age, sex, nationality, family residence, and economic status.

Post-data extraction, we rigorously reviewed and coded the information, ensuring the highest standards of patient confidentiality. Statistical analysis was performed using Statistical Product and Service Solutions (SPSS), version 26.0 (IBM SPSS Statistics for Windows, Armonk, NY). Numerical data were described as mean (±SD), while frequencies (number of cases) and valid percentages represented categorical variables. We employed the chi-square and Fisher's exact tests to ascertain the association between depression, anxiety, stress, and demographic factors. A p-value less than 0.05 signified statistical significance.

The Depression, Anxiety, and Stress Scale-21-item questionnaire (DASS-21) was used to determine emotional states. The DASS-21 comprises 21 questions, with every seven questions assessing one emotional state: depression, anxiety, or stress. The depression subscale encompasses questions 3, 5, 10, 13, 16, 17, and 21. The anxiety subscale features questions 2, 4, 7, 9, 15, 19, and 20. The stress subscale includes questions 1, 6, 8, 11, 12, 14, and 18. For each subscale, the total score of the seven questions (multiplied by two) categorizes the severity, from normal to extremely severe, of depression, anxiety, or stress.

## Results

The study included 44 participants diagnosed with one of the inherited bleeding disorders: hemophilia A or B or VWD. The mean age of the participants was 17.5 (±7.4). A substantial majority, 95.5%, hailed from Saudi Arabia, and 88.6% resided in Al-Madinah City. Over half of the participants, 52.3%, were classified within a high economic status level. Table 1 provides an overview of the participants' demographic data. Participant responses to the DASS-21 questionnaire are comprehensively captured in Table 2.

**Table 1:** Demographic characteristics of participants (n=44)

Parameters	Category	n (%)
Nationality	Saudi Arabia	42 (95.5%)
	Syria	1 (2.3%)
	Yemen	1 (2.3%)
Region	Al-Madinah city	39 (88.6%)
	Al-Ula city	1 (2.3%)
	Yanbu city	4 (9.1%)
Economic level	High	23 (52.3%)
	Intermediate	7 (15.9%)
	Low	14 (31.8%)

**Table 2:** Respondents’ answers to the DASS-21 questionnaire

Question	Response	n (%)
1. Found it hard to wind down	Did not apply at all	28 (63.6%)
	Applied to some degree, or some of the time	3 (6.8%)
	Applied to a considerable degree or a good part of time	7 (15.9%)
	Applied very much or most of the time	6 (13.6%)
2. Aware of dryness of the mouth	Did not apply at all	37 (84.1%)
	Applied to some degree, or some of the time	3 (6.8%)
	Applied to a considerable degree or a good part of time	3 (6.8%)
	Applied very much or most of the time	1 (2.3%)
3. Couldn’t experience any positive feelings at all	Did not apply at all	33 (75.0%)
	Applied to some degree, or some of the time	2 (4.5%)
	Applied to a considerable degree or a good part of time	4 (9.1%)
	Applied very much or most of the time	5 (11.4%)
4. Experienced breathing difficulty (e.g., excessively rapid breathing, breathlessness in the absence of physical exertion)	Did not apply at all	27 (61.4%)
	Applied to some degree, or some of the time	5 (11.4%)
	Applied to a considerable degree or a good part of time	9 (20.5%)
	Applied very much or most of the time	3 (6.8%)
5. Found it difficult to work up the initiative to do things	Did not apply to me at all	27 (61.4%)
	Applied to me to some degree, or some of the time	5 (11.4%)
	Applied to me to a considerable degree or a good part of time	6 (13.6%)
	Applied to me very much or most of the time	6 (13.6%)
6. Tended to over-react to situations	Did not apply at all	17 (38.6%)
	Applied to some degree, or some of the time	9 (20.5%)
	Applied to a considerable degree or a good part of time	13 (29.5%)
	Applied very much or most of the time	5 (11.4%)
7. Experienced trembling (e.g., in the hands)	Did not apply at all	28 (63.6%)
	Applied to some degree, or some of the time	6 (13.6%)
	Applied to a considerable degree or a good part of time	5 (11.4%)
	Applied very much or most of the time	5 (11.4%)
8. Felt nervous	Did not apply at all	20 (45.5%)
	Applied to some degree, or some of the time	5 (11.4%)
	Applied to a considerable degree or a good part of time	12 (27.3%)
	Applied very much or most of the time	7 (15.9%)
9. Worried about situations in which feeling panic and make a fool	Did not apply at all	25 (56.8%)
	Applied to some degree, or some of the time	5 (11.4%)
	Applied to a considerable degree or a good part of time	9 (20.5%)
	Applied very much or most of the time	5 (11.4%)
10. Feeling nothing to look forward to	Did not apply at all	36 (81.8%)
	Applied to some degree, or some of the time	3 (6.8%)
	Applied to a considerable degree or a good part of time	3 (6.8%)
	Applied very much or most of the time	2 (4.5%)
11. Found myself getting agitated	Did not apply at all	27 (61.4%)
	Applied to some degree, or some of the time	8 (18.2%)
	Applied to a considerable degree or a good part of time	7 (15.9%)
	Applied very much or most of the time	2 (4.5%)
12. Found it difficult to relax	Did not apply at all	33 (75%)
	Applied to some degree, or some of the time	4 (9.1%)
	Applied to a considerable degree or a good part of time	5 (11.4%)
	Applied very much or most of the time	2 (4.5%)
13. Felt downhearted and blue	Did not apply at all	33 (75%)
	Applied to some degree, or some of the time	5 (11.4%)
	Applied to a considerable degree or a good part of time	1 (2.3%)
	Applied very much or most of the time	5 (11.4%)
14. Intolerance of anything that kept me from getting on with what I was doing	Did not apply at all	25 (56.8%)
	Applied to some degree, or some of the time	8 (18.2%)
	Applied to a considerable degree or a good part of time	7 (15.9%)
	Applied very much or most of the time	4 (9.1%)
15. Felt close to panic	Did not apply at all	28 (63.6%)
	Applied to some degree, or some of the time	4 (9.1%)
	Applied to a considerable degree or a good part of time	4 (9.1%)
	Applied very much or most of the time	8 (18.2%)
16. Unable to become enthusiastic about anything	Did not apply at all	31 (70.5%)
	Applied to some degree, or some of the time	4 (9.1%)
	Applied to a considerable degree or a good part of time	7 (15.9%)
	Applied very much or most of the time	2 (4.5%)
17. Feeling that I wasn’t worth much as a person	Did not apply at all	33 (75%)
	Applied to some degree, or some of the time	3 (6.8%)
	Applied to a considerable degree or a good part of time	5 (11.4%)
	Applied very much or most of the time	3 (6.8%)
18. Feeling that I was rather touchy	Did not apply at all	25 (56.8%)
	Applied to some degree, or some of the time	5 (11.4%)
	Applied to a considerable degree or a good part of time	8 (18.2%)
	Applied very much or most of the time	6 (13.6%)
19. Awareness of the action of the heart in the absence of physical exertion (e.g., sense of heart rate increase, heart missing a beat)	Did not apply at all	32 (72.7%)
	Applied to some degree, or some of the time	3 (6.8%)
	Applied to a considerable degree or a good part of time	6 (13.6%)
	Applied very much or most of the time	3 (6.8%)
20. Feeling scared without any good reason	Did not apply at all	27 (61.4%)
	Applied to some degree, or some of the time	8 (18.2%)
	Applied to a considerable degree or a good part of time	6 (13.6%)
	Applied very much or most of the time	3 (6.8%)
21. Feeling that life was meaningless	Did not apply at all	36 (81.8%)
	Applied to some degree, or some of the time	2 (4.5%)
	Applied to a considerable degree or a good part of time	3 (6.8%)
	Applied very much or most of the time	3 (6.8%)

**Figure 1:**
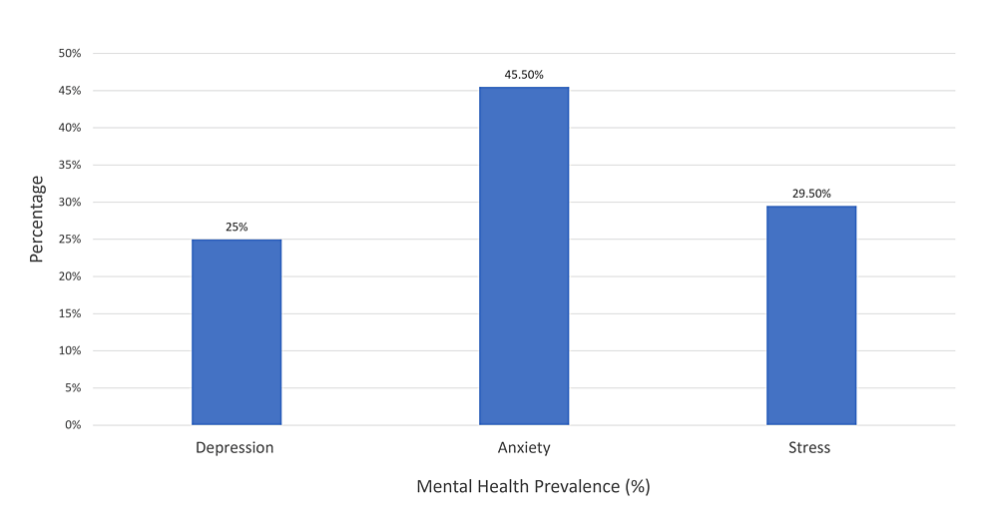
Mental health prevalence (%)

When assessing the prevalence of depression, anxiety, and stress among the participants, it was determined by aggregating the scores across the mild, moderate, severe, and extremely severe degrees. Our findings indicate that 25% of the participants exhibited symptoms of depression, 45.5% showed signs of anxiety, and 29.5% demonstrated symptoms of stress, as illustrated in Figure 1.

**Figure 2:**
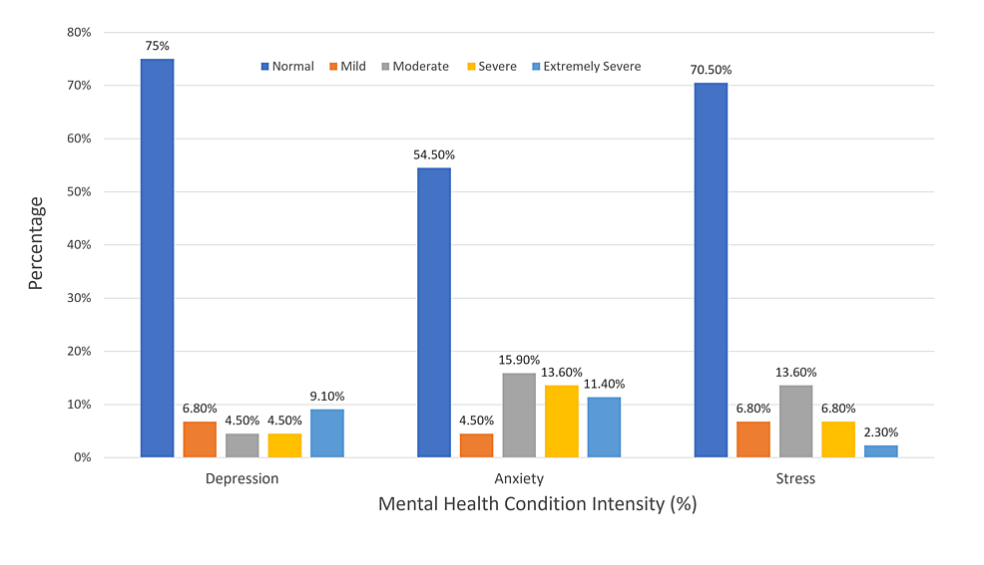
Mental health condition intensity (%)

Regarding the severity of emotional states, 9.1% of the participants reported experiencing extremely severe depression. In contrast, 15.9% were found to have a moderate level of anxiety, and 13.6% indicated a moderate stress level. Figure 2 provides a detailed breakdown of these findings.

On further examining the factors linked to the prevalence of depression, we observed that depression was significantly more common in participants over 15 years (p=0.009), as highlighted in Table 3. However, our investigation into the factors associated with anxiety prevalence, as shown in Table 4, did not reveal any statistically significant contributors affecting the prevalence of anxiety among participants. Similarly, no significant factors influencing the prevalence of stress among participants were identified, as detailed in Table 5.

**Table 3:** Association between participants’ characteristics and depression prevalence *: Significant

Factors		Depression		Total (n=44)	P-value
		Absent, n (%)	Present, n (%)		
Age	15 years old or younger	21 (91.3%)	2 (8.7%)	23	0.009*
	Older than 15 years	12 (57.1%)	9 (42.9%)	21	
Nationality	Saudi	31 (73.8%)	11 (26.2%)	42	1.00
	Non-Saudi	2 (100%)	0 (0%)	2	
Region	Al-Madinah	29 (74.4%)	10 (25.6%)	39	1.00
	Other cities	4 (80%)	1 (20%)	5	
Economic level	High	16 (69.6%)	7 (30.4%)	23	0.384
	Intermediate or low	17 (81%)	4 (19%)	21	

**Table 4:** Association between participants’ characteristics and anxiety prevalence

Factors		Anxiety		Total (n=44)	P-value
		Absent, n (%)	Present, n (%)		
Age	15 years old or younger	14 (60.9%)	9 (39.1%)	23	0.378
	Older than 15 years	10 (47.6%)	11 (52.4%)	21	
Nationality	Saudi	24 (57.1%)	18 (42.9%)	42	0.201
	Non-Saudi	0 (0%)	2 (100%)	2	
Region	Al-Madinah	20 (51.3%)	19 (48.7%)	39	0.356
	Other cities	4 (80%)	1 (20%)	5	
Economic level	High	10 (43.5%)	13 (56.5%)	23	0.123
	Intermediate or low	14 (66.7%)	7 (33.3%)	21	

**Table 5:** Association between participants’ characteristics and stress prevalence

Factors		Stress		Total (n=44)	P-value
		Absent, n (%)	Present, n (%)		
Age	15 years old or younger	18 (78.3%)	5 (21.7%)	23	0.235
	Older than 15 years	13 (61.9%)	8 (38.1%)	21	
Nationality	Saudi	29 (69%)	13 (31%)	42	1.00
	Non-Saudi	2 (100%)	0 (0%)	2	
Region	Al-Madinah	27 (69.2%)	12 (30.8%)	39	1.00
	Other cities	4 (80%)	1 (20%)	5	
Economic status	High	16 (69.6%)	7 (30.4%)	23	0.892
	Intermediate or low	15 (71.4%)	6 (28.6%)	21	

Our analysis also explored the correlation between participant characteristics and degrees of depression. Age emerged as a significant determinant influencing depression levels, with a p-value of 0.009 (Table 6).

**Table 6:** Association between participants’ characteristics and depression degrees *: Significant

Factors		Depression Degree					Total (n=44)	P-value
		Normal, n (%)	Mild, n (%)	Moderate, n (%)	Severe, n (%)	Extremely severe, n (%)		
Age	15 years old or younger	21 (91.3%)	0 (0%)	1 (4.3%)	1 (4.3%)	0 (0%)	23	0.009*
	Older than 15 years	12 (57.1%)	3 (14.3%)	1 (4.8%)	1 (4.8%)	4 (19%)	21	
Nationality	Saudi	31 (73.8%)	3 (7.1%)	2 (4.8%)	2 (4.8%)	4 (9.5%)	42	1.00
	Non-Saudi	2 (100%)	0 (0%)	0 (0%)	0 (0%)	0 (0%)	2	
Region	Al-Madinah	29 (74.4%)	2 (5.1%)	2 (5.1%)	2 (5.1%)	4 (10.3%)	39	0.636
	Other cities	4 (80%)	1 (20%)	0 (0%)	0 (0%)	0 (0%)	5	
Economic level	High	16 (69.6%)	2 (8.7%)	1 (4.3%)	2 (8.7%)	2 (8.7%)	23	0.844
	Intermediate or low	17 (81%)	1 (4.8%)	1 (4.8%)	0 (0%)	2 (9.5%)	21	

Conversely, the participant characteristics exhibited no significant impact on the degrees of anxiety or stress, as delineated in Tables 7-8.

**Table 7:** Association between participants’ characteristics and anxiety degrees

Factors		Anxiety degree					Total (n=44)	P-value
		Normal, n (%)	Mild, n (%)	Moderate, n (%)	Severe, n (%)	Extremely severe, n (%)		
Age	15 years old or younger	14 (60.9%)	1 (4.3%)	4 (17.4%)	4 (17.4%)	0 (0%)	23	0.139
	Older than 15 years	10 (47.6%)	1 (4.8%)	3 (14.3%)	2 (9.5%)	5 (23.8%)	21	
Nationality	Saudi	24 (57.1%)	2 (4.8%)	5 (11.9%)	6 (14.3%)	5 (11.9%)	42	0.084
	Non-Saudi	0 (0%)	0 (0%)	2 (100%)	0 (0%)	0 (0%)	2	
Region	Al-Madinah	20 (51.3%)	2 (5.1%)	7 (17.9%)	6 (15.4%)	4 (10.3%)	39	0.621
	Other cities	4 (80%)	0 (0%)	0 (0%)	0 (0%)	1 (20%)	5	
Economic level	High	10 (43.5%)	2 (8.7%)	3 (13%)	5 (21.7%)	3 (13%)	23	0.278
	Intermediate or low	14 (66.7%)	0 (0%)	4 (19%)	1 (4.8%)	2 (9.5%)	21	

**Table 8:** Association between participants’ characteristics and stress degrees

Factors		Stress degree					Total (n=44)	P-value
		Normal, n (%)	Mild, n (%)	Moderate, n (%)	Severe, n (%)	Extremely severe, n (%)		
Age	15 years old or younger	18 (78.3%)	2 (8.7%)	2 (8.7%)	1 (4.3%)	0 (0%)	23	0.577
	Older than 15 years	13 (61.9%)	1 (4.8%)	4 (19%)	2 (9.5%)	1 (4.8%)	21	
Nationality	Saudi	29 (69%)	3 (7.1%)	6 (14.3%)	3 (7.1%)	1 (2.4%)	42	1.00
	Non-Saudi	2 (100%)	0 (0%)	0 (0%)	0 (0%)	0 (0%)	2	
Region	Al-Madinah	27 (69.2%)	2 (5.1%)	6 (15.4%)	3 (7.7%)	1 (2.6%)	39	0.672
	Other cities	4 (80%)	1 (20%)	0 (0%)	0 (0%)	0 (0%)	5	
Economic level	High	16 (69.6%)	1 (4.3%)	2 (8.7%)	3 (13%)	1 (4.3%)	23	0.371
	Intermediate or low	15 (71.4%)	2 (9.5%)	4 (19%)	0 (0%)	0 (0%)	21	

## Discussion

Depression affects approximately 350 million people globally and is projected to become the second leading cause of debilitating illness by 2030 [6]. Patients with inherited bleeding disorders often experience psychological disturbances, influencing their treatment adherence [7]. We sought to gauge the prevalence and associated risk factors of psychological burdens, specifically depression, anxiety, and stress (DAS), in patients with inherited bleeding disorders attending hematology clinics in Madinah Province, Saudi Arabia. Our findings indicate a significant prevalence of psychological symptoms among our cohort: 25% for depression, 45.5% for anxiety, and 29.5% for stress. Breaking down by symptom severity, 9.1% of patients reported extremely severe depression, 15.9% moderate anxiety, and 13.6% moderate stress.

Recent research corroborates these high prevalence rates. A cross-sectional study noted that 33% of patients with congenital bleeding disorders experienced anxiety, 64% depression, and 60% other minor psychological complications [7,8]. An analysis of 77 patients from seven USA hemophilia treatment centers diagnosed with VWD - the most prevalent inherited bleeding disorder - revealed depression and anxiety rates of 63.6% and 58.3%, respectively [9]. In another study involving 41 adult hemophilia patients from an Arizona treatment center, 37% reported depression, with 53% describing moderate to severe depressive symptoms and 76% indicating functional impairment because of depression [9]. Similarly, a study of 200 hemophilia patients from Munson Medical Center, USA, found rates of 54% for moderate to severe depression and 52% for anxiety [10].

Most prior studies employed the Patient Health Questionnaire-9 and Generalized Anxiety Disorder 7-item scale to diagnose depression and anxiety. We opted for the DASS-21 due to its validation in Arab populations. Our region's emphasis has often been on assessing the health-related quality of life (HRQoL) among these patients to understand the impacts on various life aspects, guiding subsequent decisions. A study at King Khalid University Hospital in Riyadh found that a significant portion (89.7%) of hemophilia patients experienced knee joint issues. While treatment concerns were a prominent challenge, dimensions such as work/school, family, and social life were less affected [11].

The prevalence rates we observed varied based on patient characteristics. We noted a significantly higher depression rate in patients over age 15 (42.9% vs. 8.7%; p=0.009). This aligns with previous findings: a similar 33% rate of depression in adult hemophilia patients [7,8]. This prevalence aligns with the higher incidence of depression observed in adult hemophilia patients identified as experiencing chronic illness sequences [12]. Hence, chronically ill and adult hemophilia patients seem more susceptible to depression [13,14]. However, other studies in primary care settings reported a lower prevalence of 16% [10,11]. Such variations might stem from methodological differences, screening tools used, and differences in patient characteristics and research settings (primary vs. tertiary health care centers).

Interestingly, our findings of lower DAS rates in patients aged 15 or younger contrast with some previous studies. One study, for instance, found that VWD patients aged 12-17 years had a higher depression rate than adults aged 18 years or older (65.0% vs. 63.2%, p=0.88). The same study also found a higher anxiety rate in the 12-17-year group (70.0% vs. 53.8%, p=0.21), although the difference was not statistically significant [9]. This particular study used a different screening tool, focusing on HRQoL. The higher rate we observed for patients over 15 years might align with the 12-17-year-old age bracket of the VWD study, suggesting potential congruence between our studies.

Adolescents with inherited bleeding disorders are navigating the transition to adulthood, assuming greater self-care responsibilities. Our data showed that being unmarried was significantly associated with anxiety, underlining the value of robust social support networks for individuals with VWD. However, in the general population, earlier studies from the USA report a lower prevalence rate for depression and anxiety, ranging between 6% and 9% in younger demographics [15-17].

Though not statistically significant, we observed a variation in the prevalence of depression by patients' economic levels. Notably, rates of DAS were higher among those reporting a higher economic status. The influence of socioeconomic status (SES) on depression is well-documented, with a significant association between depression and income observed across many countries [18]. Our results, indicating a higher prevalence of DAS with increased income, align with studies from European countries [14,15]. These findings further emphasize the notable connection between depression and SES, particularly regarding educational level [19,20].

Regarding the high prevalence rate of severe DAS among patients aged 15 years or older, especially those with higher income levels, a different retrospective case-control study on hemophilia patients showed that younger patients exhibited milder depression symptoms compared to a healthy control group without hemophilia (2%) [20]. Another study found that older hemophilia patients with moderate or severe depression or anxiety symptoms were more likely to report uncontrolled pain [21]. This might be attributed to hemophilia patients' added stressors regarding future employment, potential job loss, marriage, and other life events.

This study's strengths lie in its unique contribution to the limited current research on this crucial medical topic. To our knowledge, this is the first study from Madinah Province examining the extent of psychological burdens, especially concerning patients' age and economic status. Moreover, the employment of DASS-21 for psychological disturbance screening, previously validated for the Arab population, strengthens our research methodology. DASS-21, developed by Antony et al. [22] in 1998, quantifies the severity of DAS symptoms in individuals not diagnosed with these conditions. The tool's reliability indices were 0.94 for depression, 0.81 for anxiety, and 0.92 for stress [23]. Utilizing validated tools and researcher-led data collection bolsters confidence in the acquired data's accuracy and consistency [24].

However, we must also acknowledge the study's limitations. Its cross-sectional nature precludes causal inferences. Cultural sensitivities in the study area might have made certain depression-related factors challenging to measure. Furthermore, since our sample comprises only hemophilia and VWD patients from Madinah Province, generalizing our findings to the entire inherited bleeding disorder patient population in the Kingdom or region becomes challenging. Nevertheless, our primary objective was to determine psychological burden prevalence, thereby enhancing regional literature on the topic.

## Conclusions

This study underscores the high prevalence of psychological burden in patients with inherited bleeding disorders, especially those over age 15. Acknowledging these findings in hemophilia treatment centers may elevate awareness among patients and healthcare providers. This awareness can propel the adoption of specialized screening tools, especially for high-risk patients.

Proactively screening for psychological burdens can enhance patient adherence to treatment, potentially reducing bleeding episodes and improving their quality of life. These insights should motivate hematologists and psychiatrists to design a comprehensive national multicenter study involving multiple hemophilia centers in the Kingdom. Such initiatives aim to establish robust psychological counseling services and possibly culminate in national guidelines addressing these concerns.
